# A vaccine grade of yeast *Saccharomyces cerevisiae* expressing mammalian myostatin

**DOI:** 10.1186/1472-6750-12-97

**Published:** 2012-12-19

**Authors:** Tingting Zhang, Lin Sun, Ying Xin, Lixia Ma, Youyou Zhang, Xin Wang, Kun Xu, Chonghua Ren, Cunfang Zhang, Zhilong Chen, Hanjiang Yang, Zhiying Zhang

**Affiliations:** 1 College of Animal Science & Technology, Shaan'xi Key Laboratory of Molecular Biology for Agriculture, Northwest A&F University, YangLing, Shaan'xi, 712100, China

**Keywords:** *Saccharomyces cerevisiae*, Vaccine, Myostatin, Chromosomal integration

## Abstract

**Background:**

Yeast *Saccharomyces cerevisiae* is a widely-used system for protein expression. We previously showed that heat-killed whole recombinant yeast vaccine expressing mammalian myostatin can modulate myostatin function in mice, resulting in increase of body weight and muscle composition in these animals. Foreign DNA introduced into yeast cells can be lost soon unless cells are continuously cultured in selection media, which usually contain antibiotics. For cost and safety concerns, it is essential to optimize conditions to produce quality food and pharmaceutical products.

**Results:**

We developed a simple but effective method to engineer a yeast strain stably expressing mammalian myostatin. This method utilized high-copy-number integration of myostatin gene into the ribosomal DNA of *Saccharomyces cerevisiae*. In the final step, antibiotic selection marker was removed using the Cre-LoxP system to minimize any possible side-effects for animals. The resulting yeast strain can be maintained in rich culture media and stably express mammalian myostatin for two years. Oral administration of the recombinant yeast was able to induce immune response to myostatin and modulated the body weight of mice.

**Conclusions:**

Establishment of such yeast strain is a step further toward transformation of yeast cells into edible vaccine to improve meat production in farm animals and treat human muscle-wasting diseases in the future.

## Background

Yeast *Saccharomyces cerevisiae* is a widely-used protein expression system to produce target proteins of great commercial value [1]. Such protein products include not only food or food supplements for human and animal consumption, but also varieties of non-food industrial products for the ultimate benefit of mankind.

Myostatin (MSTN), also known as growth and differentiation factor 8 (GDF8) is a member of transforming growth factor (TGF)-ß superfamily, which has been found to be capable of modulating the body weight and muscle composition in laboratory and farm animals [2-5]. In a recent proof-of-concept study, we demonstrated that oral feeding of heat-killed whole recombinant yeast *Saccharomyces cerevisiae* expressing mammalian myostatin from a plasmid elicited antigen-specific cell and humor responses, which resulted in increased body weight and muscle composition in mice [6]. Due to its simple growing method and nontoxic nature, it is reasonable to believe that such recombinant yeast can be further developed into edible vaccine, which is useful to improve meat production in farm animals and combat human muscle-wasting diseases such as muscular atrophy.

Yeast can be easily transformed with foreign DNA, which carries gene expression cassette. However, once in cells, the foreign DNA, which is often in a form of episomal plasmid, can be easily lost after a couple of rounds of cell replication unless selection pressure is continuously applied. On the other hand, to maintain the foreign DNA in cells expression of antibiotic selection marker and/or addition of antibiotics in culture may not be favorable for the purpose of protein production either for human consumption or for pharmaceutical use. Auxotrophic complementary selection markers have certain advantages over antibiotic markers; however, it remains inconvenient and costly to grow a strain with synthetic media by using these markers. To date, chromosomal integration of gene expression cassette in yeast cells offers a better choice to avoid most of the problems mentioned above. Chromosomal integration can lead to a stable expression and eliminate the need of selection pressure to maintain an established recombinant yeast strain. Here, we presented our results of engineering a yeast strain, which carries chromosomal integration of mammalian myostatin expression cassette, and stably produces myostatin protein shown by western blot. Moreover, with the help of the Cre-LoxP system we removed the selection marker gene which is absolutely nonessential for expression of target proteins.

## Results

### Construction of integration plasmids

**Figure 1:**
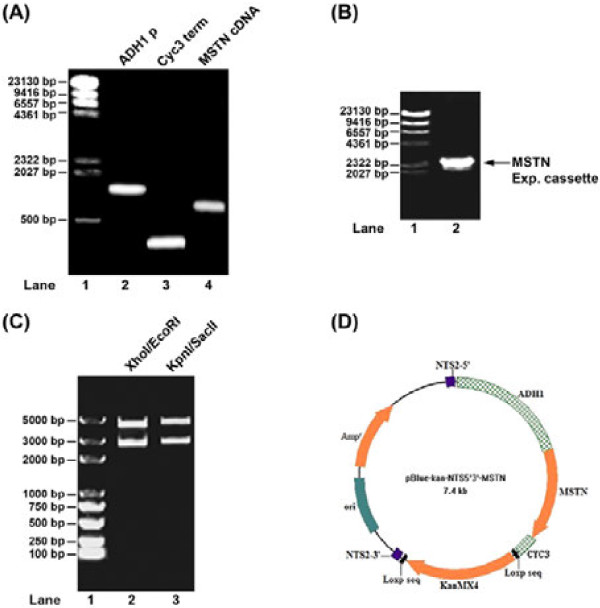
**Construction of integrate plasmid pBlue-KanMX4-NTS2-5’3’-MSTN.** (**A**) PCR amplification of ADH1 promoter (ADH1 p), Cyc3 termination signal (Cyc3 term) and the cDNA of myostatin (MSTN cDNA). (**B**) Overlapping PCR for myostatin expression cassette. (**C**) Restriction digestion of pBlue-KanMX4-NTS2-5’3’-MSTN with enzymes indicated in the figure. In panels (**A**) - (**C**), DNA fragments were separated in 1% agarose gel and visualized by ethidium bromide staining. DNA molecular weight marker was shown in lane 1. (**D**) Schematic illustration of integrate plasmid pBlue-KanMX4-NTS2-5’3’-MSTN.

The intermediate plasmids pBlue-KanMX4 and pBlue-KanMX4-NTS2-5’3’ were constructed as described in Materials and Methods. Meanwhile, we constructed myostatin expression cassette using overlap PCR strategy. To do so, PCR primers were designed to first amplify the promoter region of ADH1 gene using ADH1-F and ADH1-R primers from yeast expression vector JMB667 (a gift from Dr. Mymryk), the termination region of CYC3 gene using CYC3-F and CYC3-R primers from the same yeast expression vector as for amplification of ADH1 promoter (Table 1), the coding region of porcine myostatin cDNA using MSTN-F and MSTN-R primers from JMB88-MSTN previously described in our studies [6]and . The PCR products of these three regions were 1420 bp, 260 bp and 1128 bp, respectively (Figure 1A). After gel purification, 1 ul each of these three DNA fragments mixed with ADH1-F and CYC3-R primers were used for overlapping PCR as described in Materials and Methods. The final PCR product of myostatin expression cassette was about 2880 bp in length (Figure 1B). Then, the DNA fragment of PCR product was recovered, digested with *Xho*I and *EcoR*I, and subcloned into pBlue-KanMX4-NTS2-5’3’ on the same restriction sites. The resulting final plasmid was designated as pBlue-KanMX4-NTS2-5’3’-MSTN. The success of subcloning was confirmed by restriction digestion (Figure 1C) and by DNA sequencing (data not shown). A schematic illustration map of integration plasmid is given in Figure 1D.

**Table 1:** PCR primers (cloning sites are indicated by underscore)

**Name**	**Sequence (5’ → 3’)**
LoxP-KanMX4-F	GCGAATTCATAACTTCGTATAATGTATGCTATACGAAGTTATGTTTAGCTTGCCTCGTCC
LoxP-KanMX4-R	GCGGATCCATAACTTCGTATAGCATACATTATACGAAGTTATGTTTTCGACACTGGATGG
NTS2-5’-F	CACAAGAGGTAGGTCGAAACAGAACATGAAAGTTGGTCGGTAGGTGC
NTS2-5’-R	TCGAGCACCTACCGACCAACTTTCATGTTCTGTTTCGACCTACCTCTTGTGGTAC
NTS2-3’-F	GGCCGCCAGAGGTAGTTTCAAGGTGACAGGTTATGAAGATATGGTGCAAAACCGC
NTS2-3’-R	GGTTTTGCACCATATCTTCATAACCTGTCACCTTGAAACTACCTCTGGC
ADH1-F	GCCTCGAGAAAACAAGAAGAGGGTTGAC
ADH1-R	GTATATGAGATAGTTGATTG
CYC3-F	ATCATGTAATTAGTTATGTC
CYC3-R	GCGAATTCGCAAATTAAAGCCTTCGAGC
MSTN-F	ATCAACTATCTCATATACATGCAAAAACTGCAAATCTA
MSTN-R	CATAACTAATTACATGATTGAGCACCCACAGCGATCTA

**Figure 2:**
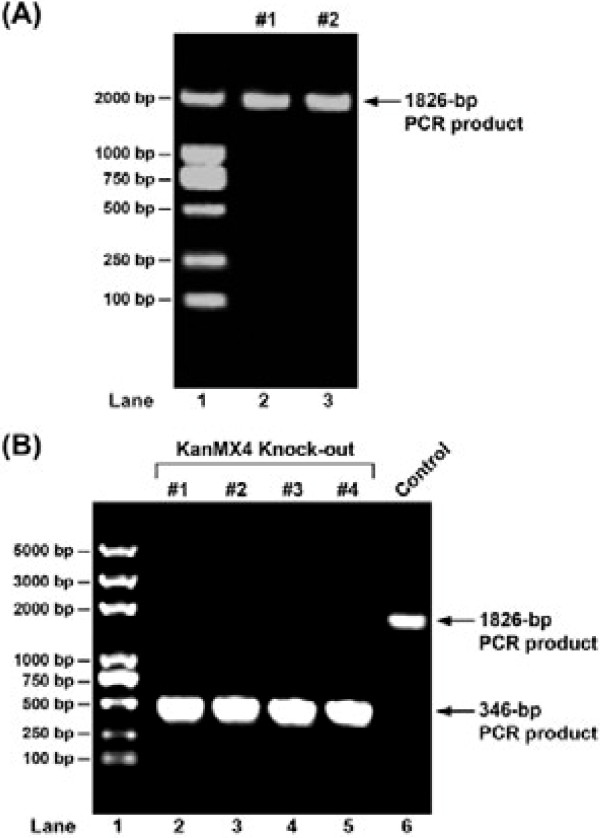
**Construction of recombinant yeast strain JMY12.** (**A**) JMY11: The success of chromosomal integration in two randomly selected yeast transformants was confirmed by PCR using primers Cyc3-F and NTS2-3’-R. (**B**) JMY12: Removal of KanMX4 expression cassette was confirmed by PCR using the same primers for identification of yeast strain JMY11. Four randomly selected yeast colonies were shown in lanes 2–4. A negative control was shown in lane 6. In both (**A**) and (**B**), DNA fragments were separated in 1% agarose gel and visualized by ethidium bromide staining. Lane 1: DNA molecular weight marker.

**Chromosomal integration of target plasmid in yeast JMY1**To integrate target plasmid into chromosomes of yeast, we followed a standard procedure of homologous DNA recombination. In brief, pBlue-KanMX4-NTS2-5’3’-MSTN plasmid was linearized by restriction digestion with *Kpn*I. Then, DNA fragment was gel-purified and used to transform yeast JMY1. Transformants were grown on YEPD rich medium agar plates containing 200 μg/ml of G418 at 30°C. Three days later, nearly a hundred positive colonies showed up on the plate, and three of them were randomly selected and amplified in fresh YEPD liquid media. Genomic DNA was extracted using the method described by Harju et al. [7]. It involved repeated freeze-thawing of cells in a lysis buffer to disrupt cell wall and membrane and release their genomic DNA. Isolated DNA was resuspended in TE buffer and further diluted to 200ug/ml. One μl of such prepared genomic DNA solution was used and subjected to PCR using CYC3-F and NTS2-3’-R primers as previously described (Table 1). Since the target plasmid had been linearized prior to transformation, it would not propagate along with cell replication, which rendered negative results for those cells without chromosomal integration. As shown in Figure 2A, two of the three colonies gave rise to an 1826-bp PCR product, indicating that the target plasmid had been successfully integrated into the chromosomes of these cells. Both colonies were then preserved in glycerol at −80°C, and one of them was designated JMY11 and used in further studies.

### Removal of KanMX4 marker gene

Regardless its molecular mechanism, retaining a selection marker gene in yeast cells would have potential deleterious effect on the production of target gene product. Towards a step further to transform yeast into edible vaccine, we decided to remove the KanMX4 selection module after the chromosomal integration of myostatin gene expression cassette was confirmed. A Cre gene expression plasmid JMB943 was transformed into JMY11, and grew in minimal SD plate depleted of uracil for 96 hours. Then, four colonies were randomly selected and tested for the removal of KanMX4 module by PCR using primers CYC3-F and NTS-3’-R (Table 1). Compared to negative control, all four colonies showed a PCR product in the size of 346 bp (Figure 2B), indicating that KanMX4 module was removed. To further confirm removal of KanMX4 module, we tested these cells on G418 selection media and did not find the cells grew on G418 media (data not shown). These colonies were preserved in glycerol solution at −80°C, and then used in further studies.

### Expression of myostatin in yeast cells

**Figure 3:**
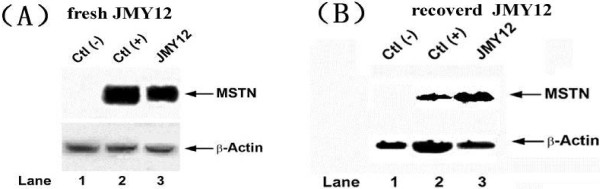
**Analysis of MSTN expression in yeast strain JMY12 by western blotting.** (**A**) A negative control from parental yeast strain JMY1 and a positive control of MSTN expression from Cu^2+^-inducible promoter were shown in lanes 1 and 2, respectively. Expression of MSTN from yeast strain JMY12 was shown in lane 3. In upper panel, MSTN expression was revealed by mouse polyclonal anti-MSTN antibody. In lower panel, western blotting for ß-actin was performed to ensure the equal loading of protein samples. (**B**) JMY12 stored for two years and recovered for the western blotting. In upper panel, MSTN expression was revealed by mouse polyclonal anti-MSTN antibody. In lower panel, western blotting for ß-actin was performed to ensure the equal loading of protein samples.

Finally, we examined the expression of recombinant mammalian MSTN in newly established yeast strain. All four colonies obtained from last step were grown in 5 ml of YEPD rich media at 30°C for 24 hours. Subsequently, yeast whole protein was extracted using LiAc/NaOH extraction methods, which was invented in our lab as previously described [8]. Western blot was carried out using home-made mouse polyclonal anti-MSTN antibody. Meanwhile, we used yeast cells expressing MSTN from a plasmid with Cu^2+^-inducible promoter as positive control. As shown in Figure 3A, a colony that carried chromosomal integration of MSTN gene and stably expressed MSTN protein had similar expression efficiency as that of inducible promoter. The strain derived from this colony was designated JMY12 and used in further studies. All other colonies expressed MSTN to similar levels with little differences (data not shown).

To test the stability of newly established yeast strain JMY12 which kept at −80°C for 2 years, we first recovered the strain JMY12 and cultured the cells in large scale for the strain stability test and oral immunization. After starting immunization experiment, we had maintained the recombinant strain in rich media for 6 months and then checked the stability every 12 hours for 10 days. Compared to protein samples previously prepared, our results showed that expression of recombinant mammalian myostatin in JMY12 was stable after long period of culture in rich media (Figure 3B).

### Effect of whole recombinant yeast vaccine on the body weight of mice

**Figure 4:**
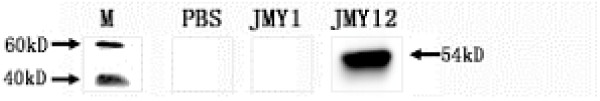
**Detection of myostatin-specific antibodies in mice serum.** A representative western blot from three independent experiments (serum samples from group PBS, JMY1, JMY12, respectively) was shown. Serum dilution was 1:2000.

The vaccination procedure carried out in this study lasted a total of 5 weeks, but the mice were maintained for additional 7 weeks before they were sacrificed. We first examined the immunization response by detection of anti-MSTN antibody in mouse serum. As shown in Figure 4, the mice immunized with the yeast strain stably expressing MSTN generated specific anti-MSTN antibody in their serum, but not detected in control group. We then examined the body weight of each animal both before and after the experiment. As shown in Table 2, when the experiment started all mice were about the same size with little difference. At the end of experiment, however, they weighted from 39 to 42 g and the average sizes varied among groups. In group PBS and group JMY1, the average increase of body weight was 16.916 ± 0.096 or 16.856 ± 0.177 g, respectively, whereas it was 19.326 ± 0.428 g in group JMY12, suggesting that vaccination against myostatin modulated the body weight of animals and oral administration of whole recombinant yeast vaccine was effective.

**Table 2:** **Effect of immunization on the body weight of mice**^**a**^

**Group**	**Before Immunization**	**After Immunization**	**Increase**
PBS	22.926 ± 0.073	39.842 ± 0.862	16.916 ± 0.096
JMY1	22.864 ± 0.696	39.720 ± 0.784	16.856 ± 0.177
JMY12^+^	22.354 ± 0.341	41.680 ± 0.768	19.326 ± 0.428*

a Mean ± standard deviation (g).

* Statistically significant difference (P < 0.05).

## Discussion

In this study, we engineered a yeast strain from *Saccharomyces cerevisiae*, which carries chromosomal integration of mammalian myostatin expression cassette and is capable of stably expressing the target protein in rich culture media without the need of maintaining selection pressure. An ideal yeast vaccine candidate should be free of antibiotic selection marker genes and easily for growing in rich media without selection pressure. We constructed such recombinant yeast strain, which could be used for a large scale preparation of edible vaccine for animals based on the functional analysis of mammalian myostatin in our previous report [6].

To ensure the expression of target protein permanently, we chose the non-transcriptional spaces of yeast rRNA gene as chromosomal integration sites. Alternatively, chromosomal integration might be also achieved by using homologous sequences to yeast chromosomal centromere or telomere regions [9]. However, the copy numbers of these integration sites are often limited to one per cell. Recent studies have shown that the non-transcribed spacers, NTS1 and NTS2, in the 5’ and 3’ end flanking regions of 5S ribosomal DNA, which separate from its upstream and downstream ribosomal RNA precursor-encoding genes are the ideal sites for chromosomal integration [5,10]. They are highly conservative and each offers approximately 150 copies per cell in yeast *Saccharomyces cerevisiae*. The actual copy number of myostatin expression cassette in our yeast can be examined by Southern blotting or in situ hybridization, but it was not determined in this study due to the lack of appropriate lab equipments and technique. Nonetheless, western blotting analysis provided a satisfactory result when compared to a MSTN-plasmid expressing strain, which was previously established and used in our lab (Figure 3A). And the new strain could stably express MTSN in rich culture after stored for two years (Figure 3B). We confirmed that similar levels of expression of MSTN to this yeast were capable of giving rise to significant immune response in mice and subsequently affected their body weight.

The primary purpose of this study was to generate a yeast strain, which stably expresses mammalian myostatin in rich media. During the construction of integration plasmid, we designed the intermediate vector pBlue-KanMX4-NTS2-5’3’ in such a way that it may serve as a unique chromosomal integration cloning vector for other target proteins. The cloning sites are commonly used restriction enzymes and can be further modified, if necessary, for any cDNA subcloning task. Thus, this vector should be applicable to most protein expression in yeast *Saccharomyces cerevisiae* and it is available for other researchers on request.

## Conclusions

The present study provides a convenient strategy to establish chromosomal integration of gene expression cassette in yeast cells. The method is simple and easy to perform. It combines previous research by others on chromosomal integration and removal of KanMX4 selection cassette by Cre-LoxP procedure. The target gene was mammalian myostatin whose expression was quite successful and those recombinant yeast cells can induce immune responses to myostatin by oral route, resulting in increasing body weight in mice. It is an important step towards transforming yeast cells into edible vaccine to improve meat production in farm animals and combat muscle-waste genetic diseases in human.

## Methods

### Animals

A total of 15 five-week-old male Kunming mice were purchased from the Breeding and Research Center of Xi'an Jiaotong University, China. Sterilized water and food were provided *ad libitum*. All animal procedures were performed in accordance with the policy and regulations for care and use of laboratory animals. The animal experimental protocols were approved by the ethic committee of Northwest A&F University before the start of the experiments.

### Yeast strain, media and culture conditions

A yeast *Saccharomyces cerevisiae* lab strain, JMY1 [MAT**α**, *his3*-Δ *1 trp1-289 rad1*-Δ *ura3-52* was routinely cultured at 30°C in YEPD medium containing 1% (w/v) yeast extract, 2% (w/v) peptone, 2% (w/v) glucose. A derivative of yeast strain JMY1 containing the vector that expresses Cu^2+^-inducible myostatin was described in precious study [6]. In this strain, expression of myostatin protein was induced by adding 0.5 mM of copper sulphate in culture media when the optical density (OD_600_) of cells reached 0.5 and continued growing for 10 hours.

### Yeast transformation

Plasmid vectors were introduced into yeast cells with LiAc method as described by Gietz and Woods [11]. In the present study, yeast transformants were immediately selected on YEPD rich media plate supplemented with 200 μg/ml of G418.

### Construction of integration plasmids

The antibiotic marker heterologous dominant KanMX4 module was chosen as the marker for the initial selection of transformation in yeast cells. First, we used the genomic DNA of Yeast Homozygous Diploid Pools (Cat#95401.H1Pool, Invitrogen, USA) as PCR template and amplified a KanMX4 expression module 1526 bp in length using primers LoxP-KanMX4-F and LoxP-KanMX4-R. The primer sequences are shown in Table 1. Then, the DNA fragment of PCR product was recovered, digested with *EcoR*I and *BamH*I, and subcloned into pBluescript II SK (+) at the same restriction sites. The resulting plasmid was designated as pBlue-KanMX4.

To increase the chance and copy number of target gene integrated into yeast chromosomes, we chose non-transcriptional space 2 (NTS2) of yeast ribosomal RNA (rRNA) gene as chromosomal integration sites. In this regard, NTS2 region was added as the homologous arms flanking MSTN expression cassette. To do so, DNA oligomers were designed to cover the 5’ end (NTS2-5’-F and NTS2-5’-R) and 3’ end (NTS2-3’-F and NTS2-3’-R) of NTS2 region, respectively (Table 1). A pair of DNA oligomers were annealed together carrying sticky ends for subcloning into pBlue-KanMX4 plasmid at corresponding restriction sites, i.e. *Kpn*I and *Xho*I for NTS2-5’, and *Not*I and *Sac*II for NTS2-3’ ends. The resulting plasmid was designated as pBlue-KanMX4-NTS2-5’3’.

### Construction of MSTN expression cassette via overlapping PCR

Prior to construction of myostatin expression cassette, ADH1 promoter, CYC3 termination signal and the coding sequence of MSTN cDNA were amplified by standard PCR as previously described [6]. Each PCR fragment was gel purified and measured for DNA concentration. Next, 10 μM of each DNA segment were mixed with DNA polymerase and dNTP in 1× reaction buffer, and pre-incubated at 94°C for 5 min. Then, the reaction was programmed at 94°C for 30 s, followed by annealing at 64°C for 30 s and primer extension at 72°C for 3 min in initial cycle, and repeated for a total of 15 cycles. In each consecutive cycle, the annealing temperature was reduced 1°C. Subsequently, the program was set at 94°C for 30 s, 50°C for 30 s and 72°C for 3 min for a total of 25 cycles. Finally, the reaction was extended at 72°C for 10 min. PCR product was gel-purified and inserted into pBlue-KanMX4-NTS2-5’3’ at the site of *Xho*I and *EcoR*I to obtain the final plasmid pBlue-KanMX4-NTS2-5’3’-MSTN.

### Western blotting

To examine myostatin expression in yeast, 5 ml yeast culture was collected and whole yeast cell extracts were prepared by LiAc/NaOH extraction method [8]. Protein samples were separated by SDS-polyacrylamide gel electrophoresis (PAGE) and transferred onto nitrocellulose membranes (Millipore, USA). Then, membranes were incubated first with mouse polyclonal anti-myostatin antibody, and then horseradish peroxidase (HRP)-conjugated goat anti-mouse IgG antibody. To ensure equal loading of protein samples, mouse monoclonal anti-ß-actin antibody was used for re-probe of the same membrane for myostatin blotting. Signals were visualized by using enhanced chemiluminescence (ECL, BoShiDe, China).

### Immunization

Mice were divided into 3 groups with 5 individuals in each group. The immunization protocol for groups was as follows: mice in group PBS were orally fed with 100 μL of PBS weekly for 5 weeks and mice in group JMY1 and JMY12 were orally fed with 1.2 × 10^8^ heat-killed whole recombinant yeast cells in 100 μL PBS weekly for 5 weeks. After immunization, all mice were maintained for additional 7 weeks. The whole experiment lasted 12 weeks. The body weight of mice was recorded weekly and serum samples were collected before immunization and at the end of the experiment, and used to determine the immune response with western blot.

## Competing interests

The authors declare that they have no competing interests.

## Authors’ contributions

TZ, LS, HY and ZZ designed this study. TZ, LS, and YX performed molecular cloning, yeast culture and western blot. YZ and LM participated in mice feed. The manuscript was prepared by TZ, LS and HY. All authors read and approved the final manuscript. XW, KX, CR, CZ, and ZC participated in preparation of reagents.

## References

[B1] Romanos MA Scorer CA Clare JJ Foreign gene expression in yeast: a review Yeast 1992 8 6 423 488 10.1002/yea.320080602 1502852

[B2] Dunner S Charlier C Farnir F Brouwers B Canon J Georges M Towards interbreed IBD fine mapping of the mh locus: double-muscling in the Asturiana de los Valles breed involves the same locus as in the Belgian Blue cattle breed Mamm Genome 1997 8 6 430 435 10.1007/s003359900462 9166589

[B3] Grobet L Martin LJ Poncelet D Pirottin D Brouwers B Riquet J Schoeberlein A Dunner S Menissier F Massabanda J A deletion in the bovine myostatin gene causes the double-muscled phenotype in cattle Nat Genet 1997 17 1 71 74 10.1038/ng0997-71 9288100

[B4] McPherron AC Lee SJ Double muscling in cattle due to mutations in the myostatin gene Proc Natl Acad Sci USA 1997 94 23 12457 12461 10.1073/pnas.94.23.12457 9356471 PMC24998

[B5] Wery J Gutker D Renniers AC Verdoes JC van Ooyen AJ High copy number integration into the ribosomal DNA of the yeast Phaffia rhodozyma Gene 1997 184 1 89 97 10.1016/S0378-1119(96)00579-3 9016957

[B6] Zhang T Yang H Wang R Xu K Xin Y Ren G Zhou G Zhang C Wang L Zhang Z Oral administration of myostatin-specific whole recombinant yeast Saccharomyces cerevisiae vaccine increases body weight and muscle composition in mice Vaccine 2011 29 46 8412 8416 10.1016/j.vaccine.2011.08.007 21840360

[B7] Harju S Fedosyuk H Peterson KR Rapid isolation of yeast genomic DNA: Bust n' Grab BMC Biotechnol 2004 4 8 10.1186/1472-6750-4-8 15102338 PMC406510

[B8] Zhang T Lei J Yang H Xu K Wang R Zhang Z An improved method for whole protein extraction from yeast Saccharomyces cerevisiae Yeast 2011 28 11 795 798 10.1002/yea.1905 21972073

[B9] Hohmann S A region in the yeast genome which favours multiple integration of DNA via homologous recombination Curr Genet 1987 12 7 519 526 10.1007/BF00419561 2834099

[B10] Klabunde J Diesel A Waschk D Gellissen G Hollenberg CP Suckow M Single-step co-integration of multiple expressible heterologous genes into the ribosomal DNA of the methylotrophic yeast Hansenula polymorpha Appl Microbiol Biotechnol 2002 58 6 797 805 10.1007/s00253-002-0957-0 12021801

[B11] Gietz RD Woods RA Genetic transformation of yeast Biotechniques 2001 30 4 816 820 822–816, 828 passim 11314265 10.2144/01304rv02

